# Assessment of stess and rest perfusion in patients early after first anterior STEMI patients treated successfully with pPCI

**DOI:** 10.1186/1532-429X-11-S1-O38

**Published:** 2009-01-28

**Authors:** Dorota Piotrowska-Kownacka, Lukasz Kownacki, Grzegorz Opolski, Leszek Krolicki, Olgierd Rowinski

**Affiliations:** Medical University of Warsaw, Warsaw, Poland

**Keywords:** Myocardial Perfusion, Stress Perfusion, Microvascular Obstruction, Delay Enhancement, Perfusion Deficit

## Introduction

Restoration of normal epicardial coronary flow in acute ST segment elevation myocardial infarction does not ensure adequate perfusion at the myocardial tissue level. In the era of primary PCI, patients with acute myocardial infarction treated successfully with PCI are discharged mostly within first week. There are no studies available regarding blood flow at the tissue level in these patients during effort.

## Purpose

Assessment of stess and rest perfusion defects early after STEMI successfully treated with pPCI.

## Methods

61 patients with first anterior STEMI (57 ± 10 yrs. 52 M) who underwent successful pPCI have been included into the study. CMR was performed on 1.5 T scanner between 5 and 10 days after pPCI. Myocardial perfusion was assessed at rest and in stress condition during infusion of adenosine (140 μg/kg b.w./min., 3 minutes infusion) during first-pass perfusion imaging. Microvascular obstruction regions (MVO) were assessed on early enhancement images acquired 1–2-minutes after stress perfusion. Delayed enhancement (DE) images were acquiered 15 minutes after Gd-DTPA. Transmurality of myocardial perfusion defecits at rest and in stress condition, MVO were evaluated using 5 point scale in 16 segments. DE was also evaluated in segment 17. The sum of scores were calculated for each variable. Scar size and MVO were additionally quantitatively analyzed using MASS software. The results were given in ml and in % of LV volume.

## Results

Only in 2 patients there was no evidence of at least subendocardial stress perfusion deficit. Stress perfusion sum of scores discriminated patients with normal EF (mean 59 ± 1%) and LV dysfunction (mean EF = 38 ± 9,7). Median stress perfusion sum of scores was 15 points (ranged 0 to 37). Median rest perfusion scores was significantly lower (3 points; ranged 0–27). Median MVO and DE sum of scores were 3 points (ranged 0–33) and 25 (3–42) respectively. DE measured as a % of LV volume has discriminated patients with severe (mean EF 31,7 ± 8,7) vs moderate LV dysfunction (mean EF 41,9 ± 9,5; p = 0,001) with a cut of point 40%. Regression model revealed that only DE and stress perfusion sum of scores have significantly contributed to the LV EF model at discharge.

## Conclusion

Despite TIMI 3 flow in coronary artery myocardial perfusion defects at the tissue level are very frequent. Only in patients with preserved LV function pharmacological stress have not induced or intensed perfusion deficits. All patients with STEMI anterior successfully treated with pPCI, even with mild LV dysfunction should avoid effort which could induce ischemia. The pathogenesis remains unknown. Further studies are needed.

